# A deterministic quantum dot micropillar single photon source with >65% extraction efficiency based on fluorescence imaging method

**DOI:** 10.1038/s41598-017-13433-w

**Published:** 2017-10-25

**Authors:** Shunfa Liu, Yuming Wei, Rongling Su, Rongbin Su, Ben Ma, Zesheng Chen, Haiqiao Ni, Zhichuan Niu, Ying Yu, Yujia Wei, Xuehua Wang, Siyuan Yu

**Affiliations:** 1 State Key Laboratory of Optoelectronic Materials and Technologies, School of Electronics and Information Technology, School of Physics, Sun Yat-sen University, Guangzhou, 510275 China; 2 Photonics Group, Merchant Venturers School of Engineering, University of Bristol, Bristol, BS8 1UB UK; 3 State Key Laboratory of Superlattices and Microstructures, Institute of Semiconductors, Chinese Academy of Sciences, P.O. Box 912, Beijing, 100083 China; 4 Synergetic Innovation Center of Quantum Information and Quantum Physics, University of Science and Technology of China, Hefei, Anhui 230026 China

## Abstract

We report optical positioning of single quantum dots (QDs) in planar distributed Bragg reflector (DBR) cavity with an average position uncertainty of ≈20 nm using an optimized photoluminescence imaging method. We create single-photon sources based on these QDs in determined micropillar cavities. The brightness of the QD fluorescence is greatly enhanced on resonance with the fundamental mode of the cavity, leading to an high extraction efficiency of 68% ± 6% into a lens with numerical aperture of 0.65, and simultaneously exhibiting low multi-photon probability (g^(2)^(0) = 0.144 ± 0.012) at this collection efficiency.

## Introduction

Bright and indistinguishable single photons are one of key elements in photonic quantum technologies^1,2^, such as quantum teleportation^3,4^, optical quantum networks^5^, and Boson sampling devices for intermediate quantum computing tasks^6–8^. In recent years, single self-assembled quantum dots (QDs) integrated into photonic microstructures^9^, including microcavities^10,11^, microlens^12^, waveguides^13^, gratings^14^ and nanowires^15,16^, have turned out to be very promising candidates for realizing bright single photon sources. An extraction efficiency in excess of 70% has been demonstrated both with micropillar^17,18^ and nanowire^15^ systems. The indistinguishability of the emitted photons, which is an equally important characteristic of single photon sources, can be achieved using resonant fluorescence excitation^19,20^. So far, bright and indistinguishable single photons are mostly achieved in QD-micropillar systems due to the large Purcell effect^21^ and the excellent suppression for the resonant laser^22,23^.

However, earlier QD-micropillar devices were based on statistical approaches by which several thousands of devices were fabricated. The yield of appropriate QDs that match optical modes both in space and spectra was in the low 10^−3^. Thus the most challenge stems from the random nature of the QD nucleation process. Considerable efforts have been devoted to deterministically embed a single, pre-selected quantum emitter in a photonic structure^11,12,14,24–27^. Most of these techniques rely on cryogenic optical lithography that can only determine circular pillars. Recently, a fast, high-throughput and wide-filed quantum dot positioning technique has been developed to locate single quantum dots with an accuracy of several nanometers^14,28^. Most attractively, it is compatible with room-temperature high-resolution electron-beam lithography, which can be used to define precise and sophisticated features such as elliptical micropillars^29^ and integrated light sources based on micropillar cavities^30^ in the future.

Here we first use the photoluminescence imaging technique developed in ref.^14^ to determine the position of single QDs in planar distributed Bragg reflector (DBR) cavities with respect to fiducial alignment marks with an average position uncertainty of ≈20 nm. We also use this information to fabricate and demonstrate QD single-photon sources in micropillar cavities. Fine tuning of the QD line into the cavity resonance is obtained at temperatures ranging from 4 K to 40 K with a device yield of approximately 45% in 47 devices. The device simultaneously exhibits high collection efficiency of 68% ± 6% into a lens with numerical aperture of 0.65, and low multi-photon probability (*g* ^(2)^(0) = 0.144 ± 0.012) at this collection efficiency.

## Results

### Determining QD’s position using photoluminescence imaging technique

**Figure 1:**
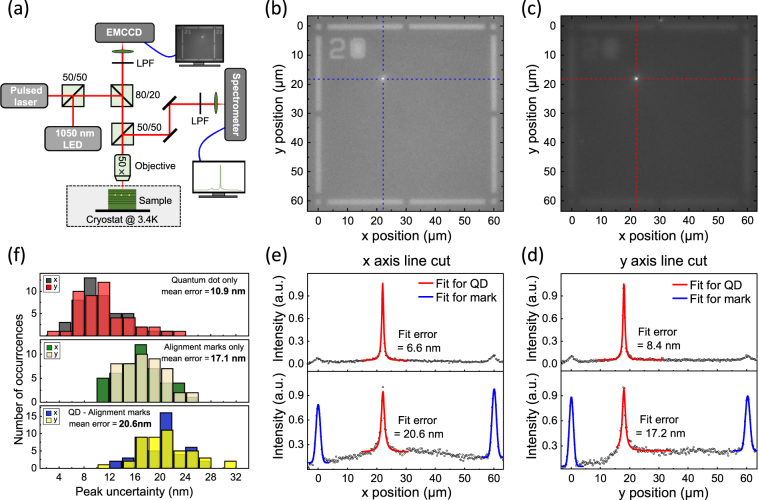
(**a**) Schematic of the micro-photoluminescence measurement and two-color photoluminescence imaging setup. (**b**–**e**) Method to acquire the relative position of the QD: (**b**) EMCCD image of the alignment marks when focusing on the surface. (**c**) EMCCD image of the photoluminescence from a single QD when focusing on the QD layer that is at the center of λ-GaAs cavity (≈1.85 *μm* below the surface). (**d**,**e**) *x*(*y*) axis line cut along the horizontal(vertical) dot line in (**b**) and (**c**), showing the QD emission, light intensity reflected by metallic marks. Herein, the Lorenz fit (red lines) and Gaussian fits (blue lines) are used to determine the location of the QD and the center position of alignment mark, respectively. The positions are then translated from a pixel value on the images to a distance on the sample by counting the number of pixels between two nearby marks with known distance. (**f**) Histograms of the uncertainties of the QD and alignment mark positions and QD-alignment mark separations (47 images). The uncertainties represent one standard deviation values determined by a nonlinear least squares fit of the data.

The deterministically positioned QD-in-micropillar structures are processed by two-color photoluminescence (PL) imaging (Fig. 1(a)) combined with standard electron-beam lithography. First, we select the QDs with their emission wavelengths near the cavity mode by collecting their emissions with a microscope objective (NA = 0.65) into a grating spectrometer. Subsequently, spatial selection is achieved by imaging the QD positions with respect to alignment marks, which is incorporated into the same micro-PL set-up. This step ensures the position of the QD at the maximum of the pillar fundamental mode.

In detail, an array of Ti/Au metal alignment marks is fabricated on the surface of the DBR planar cavity structure through a standard lift-off process. Then a wavelength-tunable pulsed laser is used to give rise to a PL emission from the QDs, while a 1050 nm light emitting diode (LED) with a power of ≈2 mW is simultaneously used to illuminate the alignment marks. The illumination wavelength of LED is chosen out of the stopband (870–980 nm) of our Bragg mirrors to regain contrast in the image. The microscope objective is focused on the QD layer at the center of λ-GaAs cavity (≈1.85 *μ*m below the surface) when imaging the fluorescence from the QDs, while imaging of the alignment marks is done by focusing on the planar surface of the structure to ensure its positioning accuracy. The exposure time of EMCCD is set at 0.1 s to reduce sample drift during images acquisition.

Representative images of the alignment marks (focused on the planar surface) and QD photoluminescence (focused on ≈1.85 *μ*m below the surface) are shown in Fig. 1(b,c), respectively. A circular bright spot and related alignment marks are clearly visible in Fig. 1(b), which represents the emission from one single QD within an ≈60 *μ*m ×60 *μ*m field of view. Orthogonal line cuts of the alignment marks are fitted with Gaussian functions using a nonlinear least squares approach, determining their centre positions with an typical uncertainty of ≈18.4 nm (Fig. 1(d,e)). While the circular spot becomes optimally focused at the cost of fading the alignment marks in Fig. 1(c), with the extracted peak of *x*-positions with one standard deviation uncertainty as low as 6.6 nm, much better than that of 20.6 nm when focused on the planar surface (Fig. 1(e)). Furthermore, histograms of the measured values in Fig. 1(f) show that the mean uncertainties in the quantum dot, alignment mark, and the QD-alignment mark separation are 10.9 nm, 17.1 nm, and 20.6 nm, respectively. Thus this PL imaging technique allow us to determine the QD position by pointing the maximum of the QD emission according to the two-dimensional alignments marks, with an average position uncertainty of ≈20 nm.

### Spectral matching by carefully designing the pillar radius

**Figure 2:**
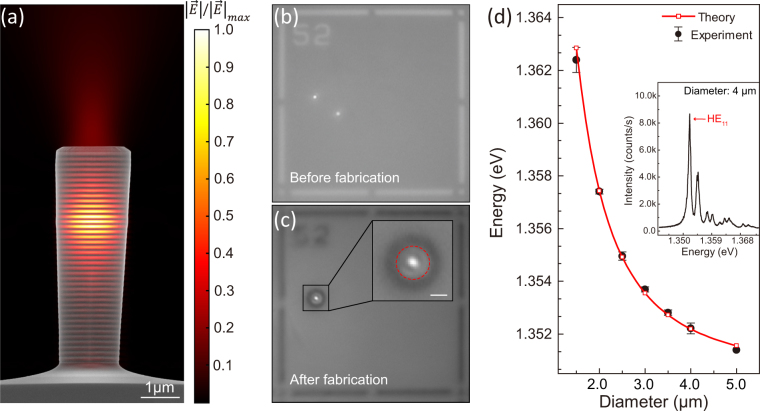
(**a**) Scanning electron microscopy (SEM) image of a typical pillar with a diameter of 2 *μ*m, along with the normalized electric field intensity distribution $|\overrightarrow{E}|$ calculated by 3D-FDTD method. (**b**,**c**) Photoluminescence images of a 4 *μ*m diameter micropillar with a single quantum dot in the center before (**b**) and after (**c**) fabrication. Scale bar represents 2 *μ*m. (**d**) The average of measured energy (black dot with error bar) of the fundamental mode (HE _11_) for the pillar cavities as a function of the designed diameter, which are well described by theory according to Eq. (1) plotted in red line. Inset is a typical experiment cavity mode of a micropillar with a diameter of 4 *μ*m acquired by raising the excitation power.

After selecting the QD with desired photon energy (around the planar cavity mode) and accurately determining its position, the fundamental mode of pillar should be carefully designed to achieve spectral matching. As the energy of fundamental mode increases when the radius decreases^31,32^, the pillar radius (R) is purposefully chosen according to the deviation of the emission frequency of the QD from the planar cavity mode. And then, typical micropillar cavities are fabricated (see micropillar fabrication in the Method Section). A scanning electron microscopy (SEM) image of a typical pillar with a diameter of 2 *μ*m is presented in Fig. 2(a), which is superimposed with the normalized electric field intensity distribution ($|\overrightarrow{E}|$) calculated by 3D-FDTD method (detailed in Fig. S1 in Supplementary Information). Figure 2(b),(c) show the representative photoluminescence images of the device before and after fabrication, indicating a QD emission is just in the center of a micropillar structure. Figure 2(d) presents the measured and theoretical energy of the fundamental mode for the pillar cavities as a function of the designed diameter. The black circles represent the experiment cavity modes of different diameters acquired by raising the power of excitation laser, which is well matched to the theory result (red line) according to equation^32,33^:

1 $$E=\sqrt{{E}_{2D}^{2}+\frac{{\hslash }^{2}{c}^{2}}{\varepsilon }\frac{{\chi }_{{n}_{\phi },{n}_{r}}^{2}}{{R}^{2}}}$$

Where ${E}_{2D}$ is the resonance of the planar cavity, ${\chi }_{{n}_{\phi },{n}_{r}}$ represents the ${n}_{r}^{th}$ zero of the Bessel function ${J}_{{n}_{\phi }(\frac{{\chi }_{{n}_{\phi },{n}_{r}}}{R})}$, and R is the radius of the pillar. For the fundamental HE _11_ mode, the quantum numbers (${n}_{\phi },{n}_{r}$, 0) is (1, 0, 0), and ${\chi }_{\mathrm{1,0}}$ equals to 2.4048 here.

By selecting appropriate pillar diameter for QD with different emission energy, we achieve a device yield of 45% in 47 devices by matching the emission wavelength between QD and fundamental mode in the range of 4 K to 40 K. The deviation from an ideal fabrication process is mainly due to the large diameter interval of 0.5 *μ*m, the slightly shifts of QD emissions during heating and cooling for several times or within the etching processes that change the strain environment of the QDs, which are also found in ref.^17^.

### Single photon source performance

**Figure 3:**
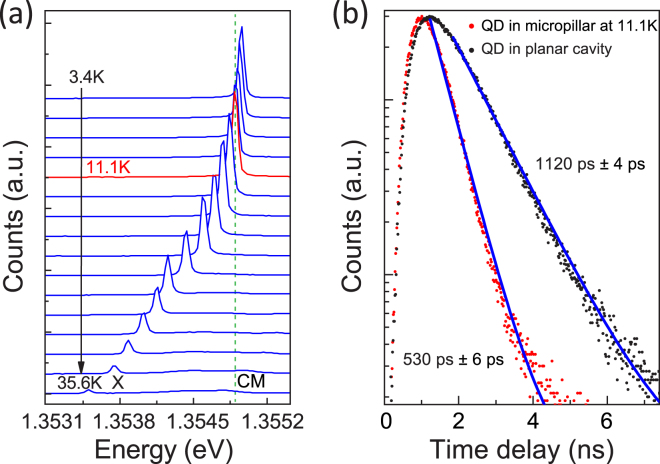
(**a**) Temperature dependent spectra of a micropillar with a diameter of 2 *μ*m, a strong enhancement on spectral resonance between fundamental mode (FM) and QD due to the Purcell effect is observed at T = 11.1 K. (**b**) Time resolved measurements of the QD under above-barrier excitation (780 nm) before fabrication (in planar structure) and in the micropillar cavity at the temperature of 11.1 K.

Now we turn to characterize the emission produced by the optically positioned QD within a micropillar with a diameter of 2 *μ*m. The extracted Q factor of fundamental mode (${Q}_{pillar}$) in our investigated device is 1438 ± 2. A typical temperature dependent micro-PL is presented in Fig. 3(a). A strong enhancement on spectral resonance between fundamental mode (FM) and QD due to the Purcell effect is observed at T = 11.1 K. Time-photoluminescence measurements are carried out to determine the Purcell enhancement of the system. The spontaneous emission decay of the QD before fabrication (in planar structure) and in the micropillar cavity (tested at 11.1 K) are shown in Fig. 3(b). The single exponential fits of the decay curves indicate a lifetime of ${\tau }_{on-resnance}$ = 530 ± 6 ps for the QD in the micropillar cavity under above-barrier excitation (780 nm) and a lifetime of ${\tau }_{0}\,=\,$ 1120 ± 4 ps for the QD in the planar structure, according to the measured lifetime, we calculate the Purcell enhancement of the spontaneous emission rate as a factor of ${F}_{p}=\frac{{\tau }_{0}}{{\tau }_{on-resnance}}=2.1\pm 0.3$, which is different from the theoretical maximum of ${F}_{p}$ = 5.4 for a micropillar with 12/25 pairs DBRs and a diameter of 2 *μ*m (Fig. S2 in Supplementary Information). The large deviation of the PL decay time might be caused by a long carrier relaxation time from higher energy states to the lowest exciton state under above-barrier excitation^34–36^ or the slow decay processes of other emitters non-resonantly coupled to the cavity, which masks the real ${F}_{p}$. The theoretical Q-factor of the planar cavity is calculated to be 1820 with 3D-FDTD method, and the experimental ${Q}_{2D}$ is extracted to be 1676 by raising the excitation power in the high density region of the wafer, a slight reduction of ${Q}_{2D}$ can be caused by the absorption in the active QDs layer^32,37^.

**Figure 4:**
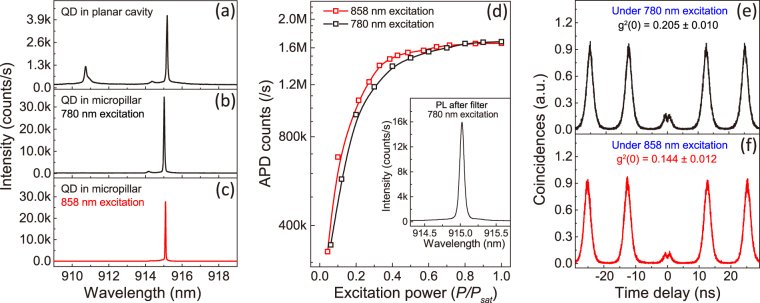
(**a**,**b**) PL spectra of a single QD in a micropillar with a diameter of 2 *μ*m before (**a**) and after (**b**) fabrication under non-resonant, 780 nm pulsed excitation. (**c**) PL spectrum of the QD in micropillar under 858 nm pulsed excitation. (**d**) Detected fluorescent counts of the same QD as a function of the normalized pulse laser power $P/{P}_{sat}$ under 780 nm (black) and 858 nm (red) pulsed excitation, here $P$ and ${P}_{sat}$ represent to the excitation and saturation power. The inset shows a spectrum after a longpass filter and a narrow band filter with a bandwidth of 1 nm. (**e**,**f**) Intensity-correlation histogram obtained using a Hanbury Brown and Twiss-type set-up under 780 nm (**e**) and 858 nm (**f**) pulsed excitation. The value of ${g}^{2}\mathrm{(0)}$ is calculated from the integrated photon counts in the zero time delay peaks divided by the average of the adjacent four peaks, and its error denotes one standard deviation. The fitting function for each peak is the convolution of a double exponential decay (exciton decay response) with a Gaussian (single-photon detector time response)^19^. Owing to the limited time response, the small two peaks around the zero time have finite overlaps.

To prove the brightness of this optical positioned QD in micropillar structure, we determine both the collection efficiency and the second order autocorrelation function at zero delay ${g}^{\mathrm{(2)}}\mathrm{(0)}$ when the QD emission is saturated. Figure 4(a,b) presents a PL spectrum of a single QD before (Fig. 4(a)) and after (Fig. 4(b)) fabrication under non-resonant,780 nm pulsed excitation. Only the emission line which has a central wavelength (915.01 nm) within cavity mode appears with bright luminescence. In order to get pure QD fluorescence, a narrow band filter with a bandwidth of 1 nm is inserted into the collection arm of the confocal optical path. The inset in Fig. 4(d) shows a spectrum after filtering in which only one peak remains. Figure 4(d) shows the detected fluorescent counts on a silicon single-photon detector as a function of normalized pulse laser power, achieving the total flux ${N}_{total}$ = 1,679,000 counts/s. To deduce the corresponding number of photons collected per excitation pulse in the first lens, we calibrate all the optical components of the detection path, as shown in Table 1. We estimate the total transmission rates of optical set-ups as ${\eta }_{setup}=\mathrm{(2.7}\pm 0.24) \%$, where the uncertainty is based on the spread of transmission values measured for the optical components, and represents a one standard deviation value. To verify that these photons are true single photon, namely only one photon is generated when QD is driven by one laser pulse, we carried out an intensity-correlation measurement at saturated pump power density of 24 W/cm^2^. The result is displayed in Fig. 4(e). Although there is a dip at zero time delay which indicates only one photon generation at a time, two obvious small peaks around zero time delay lead to a ${g}^{\mathrm{(2)}}\mathrm{(0)}$ of 0.205 ± 0.010. Here, the value of ${g}^{\mathrm{(2)}}\mathrm{(0)}$ is calculated from the integrated photon counts in the zero time delay peaks divided by the average of the adjacent four peaks, and its error denotes one standard deviation. The fitting function for each peak is the convolution of a double exponential decay (exciton decay response) with a Gaussian (single-photon detector time response)^19^. In these measurements, the proper mode-locking of the pulsed laser was carefully checked. These observations of two-peaks are not unique and occur in a similar way on a multitude of dots on this sample or other samples grown using the same MBE system^38^. We attribute these two peaks to a recapture process with assistance of trapped states in the QD sample^39,40^. The carriers can be trapped in these states first for a certain time and after that there is a recapture process from trapped states into the QD following the initial recombination^38–41^. To remove the effect of re-excitation, we multiply the total flux ${N}_{total}$ with $\frac{1}{1+{g}^{\mathrm{(2)}}\mathrm{(0)}}$ and get a pure single photon flux ${N}_{0}$. Thus we estimate extraction efficiency $\eta$ that is the percentage of generated single photons collected into the first objective lens (NA = 0.65) as $\eta$ = $\frac{{N}_{0}/{\eta }_{setup}}{79.3\,MHz}$ = 65% ± 6%. To obtain high pure combined with high brightness, we study the QD emission under 858 nm pulsed excitation (near the wetting layer of QD). There is only one peak left in the spectrum as shown in Fig. 4(c). The power dependent fluorescent counts and the intensity autocorrelation measurement presented in Fig. 4(d) and Fig. 4(f) indicate a maximum of 1,657,000 counts/s with ${g}^{\mathrm{(2)}}\mathrm{(0)}$ of 0.144 ± 0.012 at saturated pump power, revealing an extraction efficiency of 68% ± 6%. To verify such an extraction efficiency, we measured the PL intensity as a function of the energy detuning between the QD emission and cavity mode under 780 nm continuous-wave (CW) excitation^42–44^. By modeling the experimental data, we yield a Purcell factor of 3.95 ± 2.44 (details in Supplementary Information section 3), the error can be caused by the uncertainty of intensity measurements. This result agrees well with the ${F}_{p}=4.3$ calculated using 3D-FDTD simulation, and can satisfy the ${F}_{p}$ needed (around 4) to achieve an extraction efficiency of 68% ± 6% according to the well known equation $\eta =\frac{{Q}_{pillar}}{{Q}_{2D}}\times \frac{{F}_{p}}{1+{F}_{p}}\mathrm{.}$

**Table 1:** Experimental set-up calibration.

	Transmission	Error bar
Optical window	0.929	±3.0%
50 × microscope objective	0.787	±3.0%
50/50 beam splitter	0.490	±3.0%
50/50 beam splitter	0.490	±3.0%
Silver mirror	0.956	±3.0%
A 920 nm narrow band filter/a 900 nm long pass filter	0.568	±2.0%
A coupling lens	0.960	±3.0%
Single-photon detector efficiency	0.300	±5.0%
Overall detection efficiency	0.027	±9.1%

## Discussion

In this paper, we have realized positioning single QDs in planar DBR cavity with respect to alignment marks with an average position uncertainty of ≈20 nm using an optimized two-color photoluminescence imaging technique developed by Luca Sapienza and coworkers^14^. We have used this technique to create single-photon sources based on positioned QD in a micropillar cavity that simultaneously exhibit high brightness ($\eta$ = 68% ± 6%) and purity (${g}^{\mathrm{(2)}}\mathrm{(0)}=0.144\pm 0.012)$. As a next step one could also implement a resonance fluorescence excitation to achieve highly indistinguishable on-demand photons. We believe these deterministic single QD micropillar structures can be used in devices including strongly-coupled QD-microcavity systems^45,46^, on-chip quantum optics with quantum dot microcavities^30^, and orbital angular momentum modes (OAM) from quantum light sources^47^, which is very encouraging for the implementation of integrated quantum dot based quantum circuits^2^.

## Methods

### Sample growth

The investigated sample consists a single layer of low density In(Ga)As QDs grown via molecular beam epitaxy and located at the center of a λ-thick GaAs cavity surrounded by two Al _0.9_ Ga _0.1_ As/GaAs Bragg mirrors with 12 (25) pairs. The density of self-assembled InAs quantum dots varies continuously along the wafer by stopping the rotation of the substrate during InAs deposition. In our experiment, a density of about 10^8^ cm^−2^ was chosen for photoluminescence imaging. A silicon delta-doping was introduced 10 nm above the QD layer to stochastically charge the single QDs with an excess electron.

### Micropillar fabrication

The sample is first spin coated with a negative tone electron beam resist (HSQ fox15); The resist is exposed using a VISTEC EBPG5000 ES PLUS electron-beam lithography (EBL) system at 100 kV; Followed by the exposure and development process, the mask pattern of the pillar with a certain diameter is transferred into the sample via an inductively-coupled plasma reactive ion etching system (ICP-RIE, Oxford Instrument Plasmalab System 100 ICP180).

### Optical measurements

An optical microscopy cryostat (Montana, T = 4 K-300 K) mounted on a motorized positioning system with piezo-electric actuators is used for optical measurements. A 800 fs pulsed laser with tunable wavelengths from 750 nm to 1040 nm and a 79.3 MHz repetition rate is used to give rise to a PL emission from the QDs. The laser beam was focused onto a selected QD with the laser spot of ≈1.5 *μ*m. Reflected light and fluorescence from the sample go back through the 50/50 and 80/20 beam splitters and are imaged onto an Electron Multiplied Charged Couple Device (EMCCD) or a spectrometer. Two 900 nm long-pass filters (LPFs) are inserted in front of the EMCCD camera and the spectrometer respectively to remove reflected excitation light. Our auto-correlation measurements is taken out using typical Hanbury Brown and Twiss (HBT)-type set-up.

## Electronic supplementary material

Supplementary Information
