# Supplementary material for: A deterministic quantum dot micropillar single photon source with >65% extraction efficiency based on fluorescence imaging method
Source: Sci Rep. 2017 Oct 25;7:13986. doi: 10.1038/s41598-017-13433-w (PMC5656632; doi:10.1038/s41598-017-13433-w)
Supplement: Supplementary file 1 — Supplementary Information [file 41598_2017_13433_MOESM1_ESM.pdf]

A deterministic quantum dot micropillar single photon source with $> 65\%$ extraction efficiency based on fluorescence imaging method: Supplementary Information

Shunfa Liu[1], Yuming Wei[1], Rongling Su[1], Rongbin Su[1], Ben Ma[2,3], Zesheng Chen[2,3], Haiqiao Ni[2,3], Zhichuan Niu[2,3], Ying Yu*[1], Yujia Wei†[1], Xuehua Wang[1] and Siyuan Yu[1,4]

[1] *State Key Laboratory of Optoelectronic Materials and Technologies, School of Electronics and Information Technology, School of Physics, Sun Yat-sen University, Guangzhou 510275, China*

[2] *State Key Laboratory of Superlattices and Microstructures, Institute of Semiconductors, Chinese Academy of Sciences, P.O. Box 912, Beijing 100083, China*

[3] *Synergetic Innovation Center of Quantum Information and Quantum Physics, University of Science and Technology of China, Hefei, Anhui 230026, China*

[4] *Photonics Group, Merchant Venturers School of Engineering, University of Bristol, Bristol BS8 1UB, UK*

I. 3D-FDTD simulation of electric field intensity distribution

The full-vector time-dependent Maxwells equations were solved using the 3D-FDTD method on a computational grid with perfectly matched layers (PML) boundary conditions. GaAs/AlGaAs micropillar is introduced in the calculation domain with size of 5 μm$\times$ 5 μm $\times$ 13 μm. The structural parameters of the pillar are the same as referenced sample. Average refractive indices of GaAs and $\mathrm{Al}_{0.9}Ga_{0.1}As$ were 3.56 and 3.05, respectively. A dipole source in range of 5 nm (center at 918 nm) is used to represent a InAs quantum dot lying in the center of one-λ GaAs cavity. The simulation stores the time evolution of the electromagnetic fields and the intensity profile(Fig S1) is plotted for a two-dimensional cut along x-z axis in log scale, which shows that almost all the light emitted by the dipole is coupled to the pillar cavity mode and escape through the top DBR with small divergence angle.

II. The theoretical Purcell factor and Q factor of the ideal device

The theoretical calculations of Purcell factor (F_p) (Fig S2) and a Q factor for our 12/25 micropillar with a diameter of 2 μm using 3D-FDTD method are F_p=5.4 and Q=1820, respectively.

*yuying26@mail.sysu.edu.cn

†yujia.lisa.wei@gmail.com

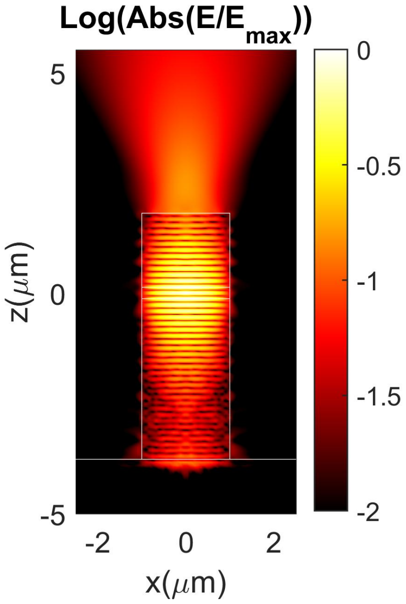

Fig S 1. Normalized two-dimensional intensity profile of the pillar cavity fundamental mode with the diameter of 2 μm along x-z axis in log scale, the white line represents the outline of pillar and cavity.

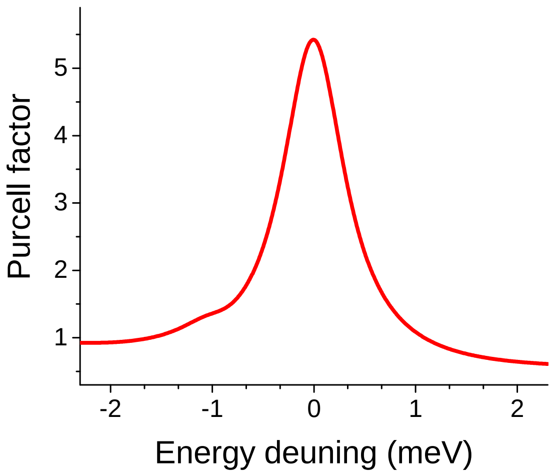

Fig S 2. Theoretical calculations of Purcell factor (F_p) and a Q factor (Q) for our 12/25 micropillar with a diameter of 2 μm using 3D-FDTD method.

III. The analysis and derivation of the real Purcell factor

As shown in last section, the theoretical Q factor and F_p of 2 μm pillar is 1820 and 5.4, respectively. In real, for our device, the Q factor of the planer cavity (Q_{2D}) is extracted to be 1676, the Q factor of the fabricated pillar with the diameter of 2 μm become 1438 due to the loss from sidewall and active layer absorption, and the F_p is derived to be 4.3 with such a Q factor compared with the theoretical one.

The F_p can be measured experimentally by two methods. The first one is to carry out time-resolved photoluminescence (PL) measurement, and compare the lifetime of a QD on and off resonant with the cavity mode. This method is intuitive and simple but may be limited by the time resolution of detector, and influenced by the capture and relaxation time of electron and holes inside the QD under non-resonant excitation. Another one is to measure the PL intensity as a function of the energy detuning between the QD emission and cavity mode, which can be carried out under continuous-wave (CW) excitation[1–3].

Fig S3 shows the integrated intensity of the QD emission under 780 nm excitation (black dot). By changing the temperature, the QD emission is detuned on and off resonant with the cavity mode. The data is fitted by the function[1]:

$$I_x(\Delta) = A \times \frac{F_p/(1+4\Delta^2/\tau_c{}^2)}{1+\sqrt{2F_p/(1+4\Delta^2/\tau_c{}^2)}}, \tag{1}$$

where A is the coefficient, Δ is the energy detuning between QD emission and cavity mode, τ_c represents the linewidth of the cavity mode, which equals to 0.94 meV for the cavity with a Q factor of 1438. By modeling the experimental data (red line), we yield a Purcell factor of 3.95±2.44, the fit error can be caused by the uncertainty of intensity measurements. This result agrees well with the $F_p = 4.3$ calculated using 3D-FDTD simulation, and can satisfy the F_p needed to achieve a collection efficiency $\eta = \frac{N_{total}}{1+g^2(0)} \frac{1}{79.3MHz} \frac{1}{\eta_{setup}}$ = 68%±6%, according to the equation[4] $\eta = \frac{Q_{pillar}}{Q_{2D}} \times \frac{F_p}{1+F_p}$.

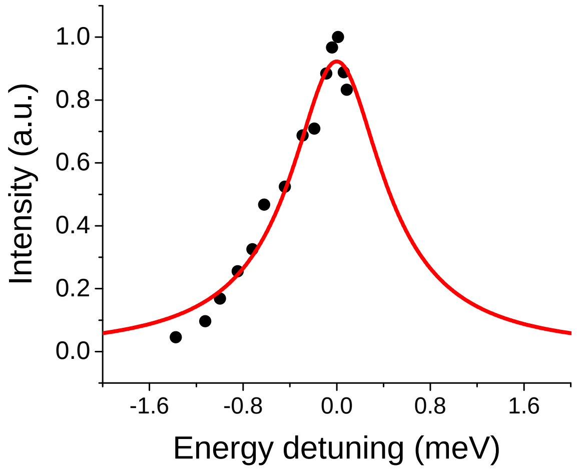

Fig S 3. Integrated intensity of the QD emission under 780 nm excitation (black dot), the pump power is significantly below the saturation of the QD. By fit the data as a function of the Energy detuning(Δ), the F_p is derived to around be 3.95 (red line).

References

[1] C Böckler, S Reitzenstein, C Kistner, R Debusmann, A Löffler, T Kida, S Höfling, A Forchel, L Grenouillet, J Claudon, et al. Electrically driven high-q quantum dot-micropillar cavities. *Applied*

Physics Letters, 92(9):091107, 2008.

[2] Mathieu Munsch, Alexis Mosset, Alexia Auffeves, Signe Seidelin, Jean-Philippe Poizat, J-M Gérard, A Lemaître, I Sagnes, and P Senellart. Continuous-wave versus time-resolved measurements of purcell factors for quantum dots in semiconductor microcavities. *Physical Review B*, 80(11):115312, 2009.

[3] Sebastian Unsleber, Christian Schneider, Sebastian Maier, Yu-Ming He, Stefan Gerhardt, Chao-Yang Lu, Jian-Wei Pan, Martin Kamp, and Sven Höfling. Deterministic generation of bright single resonance fluorescence photons from a purcell-enhanced quantum dot-micropillar system. *Optics express*, 23(26):32977–32985, 2015.

[4] WL Barnes, Gunnar Björk, JM Gérard, P Jonsson, JAE Wasey, PT Worthing, and Valéry Zwiller. Solid-state single photon sources: light collection strategies. *The European Physical Journal D-Atomic, Molecular, Optical and Plasma Physics*, 18(2):197–210, 2002.
